# 4-{[7-(Trifluoro­meth­yl)quinolin-4-yl]amino}­benzene­sulfonamide–ethanol–methanol (1/0.47/0.53)

**DOI:** 10.1107/S1600536812029698

**Published:** 2012-07-10

**Authors:** Mostafa M. Ghorab, Mansour S. Al-Said, Abdullah A. Al-Mishari, Ching Kheng Quah, Hoong-Kun Fun

**Affiliations:** a Medicinal, Aromatic and Poisonous Plants Research Center (MAPPRC), College of Pharmacy, King Saud University, PO Box 2457, Riyadh 11451, Saudi Arabia; b X-ray Crystallography Unit, School of Physics, Universiti Sains Malaysia, 11800 USM, Penang, Malaysia

## Abstract

In the title compound, C_16_H_12_F_3_N_3_O_2_S·0.47C_2_H_5_OH·0.53CH_3_OH, the quinoline ring system is approximately planar, with a maximum deviation of 0.035 (3) Å, and makes a dihedral angle of 52.67 (9)° with the benzene ring. The F atoms of the –CF_3_ group are disordered over two orientations, with refined site occupancies of 0.56 (2) and 0.44 (2). A single solvate site is occupied at random by ethanol or methanol, with refined site occupancies of 0.470 (6) and 0.530 (6), respectively. In the crystal, mol­ecules are linked *via* N—H⋯O, N—H⋯N, O—H⋯O and C—H⋯O hydrogen bonds, thereby forming sheets lying parallel to (010).

## Related literature

For background to the biological and pharmacological activity of quinolines, see: Ghorab *et al.* (2011 ▶, 2012 ▶).
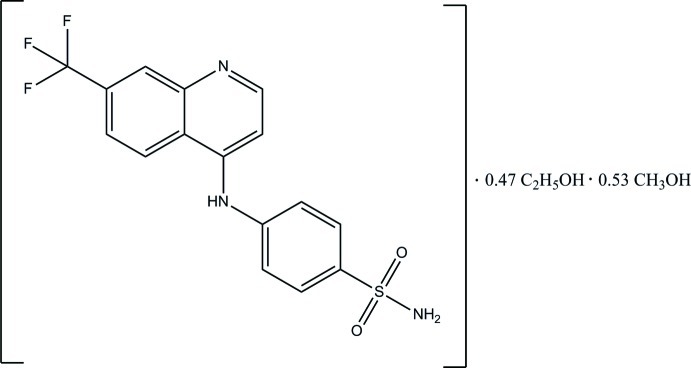


## Experimental

### 

#### Crystal data

C_16_H_12_F_3_N_3_O_2_S·0.47C_2_H_6_O·0.53CH_4_O
*M* *_r_* = 405.98
Triclinic, 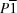

*a* = 8.6037 (1) Å
*b* = 9.3146 (2) Å
*c* = 11.4590 (2) Å
α = 92.463 (1)°
β = 91.544 (1)°
γ = 92.969 (1)°
*V* = 915.85 (3) Å^3^
*Z* = 2
Cu *K*α radiation
μ = 2.07 mm^−1^
*T* = 296 K
0.83 × 0.43 × 0.11 mm

#### Data collection

Bruker SMART APEXII CCD diffractometer
Absorption correction: multi-scan (*SADABS*; Bruker, 2009 ▶) *T*_min_ = 0.279, *T*_max_ = 0.801
9433 measured reflections
2845 independent reflections
2631 reflections with *I* > 2σ(*I*)
*R*_int_ = 0.026

#### Refinement

*R*[*F* ^2^ > 2σ(*F* ^2^)] = 0.045
*wR*(*F* ^2^) = 0.129
*S* = 1.05
2845 reflections
309 parameters
2 restraints
H atoms treated by a mixture of independent and constrained refinement
Δρ_max_ = 0.34 e Å^−3^
Δρ_min_ = −0.41 e Å^−3^

### 

Data collection: *APEX2* (Bruker, 2009 ▶); cell refinement: *SAINT* (Bruker, 2009 ▶); data reduction: *SAINT*; program(s) used to solve structure: *SHELXTL* (Sheldrick, 2008 ▶); program(s) used to refine structure: *SHELXTL*; molecular graphics: *SHELXTL*; software used to prepare material for publication: *SHELXTL* and *PLATON* (Spek, 2009 ▶).

## Supplementary Material

Crystal structure: contains datablock(s) global, I. DOI: 10.1107/S1600536812029698/hb6882sup1.cif

Structure factors: contains datablock(s) I. DOI: 10.1107/S1600536812029698/hb6882Isup2.hkl

Supplementary material file. DOI: 10.1107/S1600536812029698/hb6882Isup3.cml

Additional supplementary materials:  crystallographic information; 3D view; checkCIF report

## Figures and Tables

**Table 1:** Hydrogen-bond geometry (Å, °)

*D*—H⋯*A*	*D*—H	H⋯*A*	*D*⋯*A*	*D*—H⋯*A*
N2—H1*N*2⋯O3	0.87	2.21	3.016 (6)	153
N3—H2*N*3⋯N1^i^	0.85 (3)	2.08 (3)	2.924 (3)	169 (3)
N3—H1*N*3⋯O1^ii^	0.87 (3)	2.26 (3)	3.107 (3)	163 (3)
O3—H1*O*3⋯O1^iii^	0.96	2.49	3.387 (6)	155
O3—H1*O*3⋯O2^iii^	0.96	2.59	3.425 (6)	146
C5—H5*A*⋯O1^iii^	0.93	2.50	3.343 (3)	151
C16—H16*A*⋯O3	0.93	2.51	3.287 (6)	141

Symmetry codes: (i) 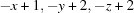; (ii) 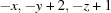; (iii) 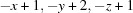.

**Fig. 1.**
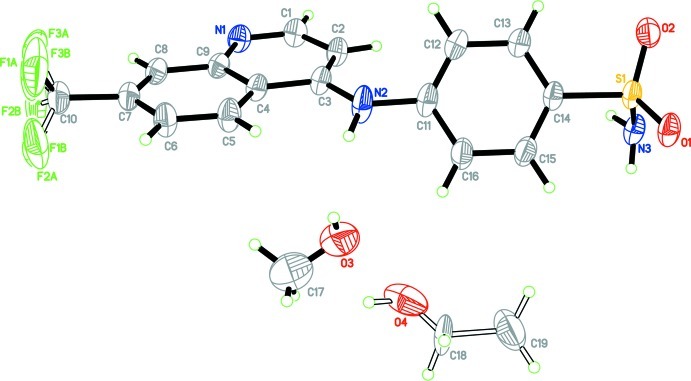
The molecular structure of the title compound showing 30% probability displacement ellipsoids for non-H atoms. Both major and minor components of disorder are shown.

**Fig. 2.**
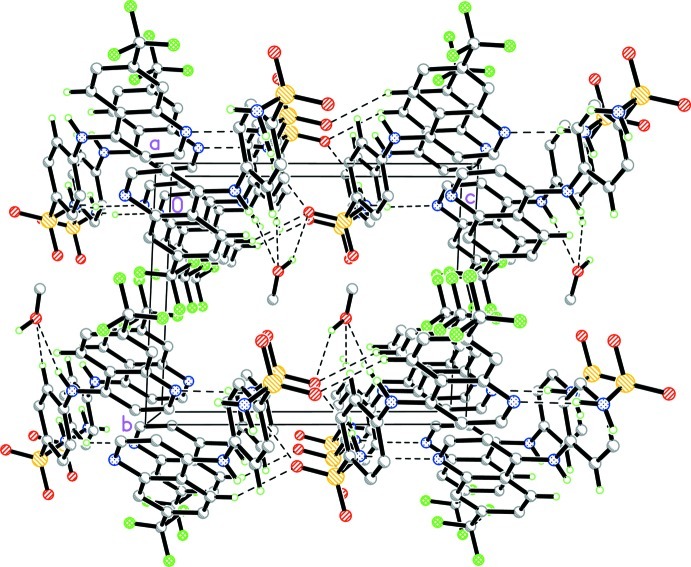
The crystal structure of the title compound, viewed along the a axis. H atoms not involved in hydrogen bonds (dashed lines) have been omitted for clarity. Only the major disorder component is shown.

## supplementary crystallographic information

### Comment

As a continuation of our efforts towards synthesizing biologically active heterocyclic compounds (Ghorab *et al.*, 2011, 2012), we have prepared the title quinoline carrying a sulfonamide moiety to evaluate its anticancer activity, which will be reported later.

In the title molecule, Fig. 1, the quinoline ring system (N1/C1-C9) is nearly planar with a maximum deviation of 0.035 (3) Å at atom C1 and it makes a dihedral angle of 52.67 (9)° with the benzene ring (C11-C16). The F atoms (F1/F2/F3) are each disordered over two positions with refined site-occupancies of 0.56 (2) and 0.44 (2). A single solvate site is occupied at random by ethanol or methanol with refined site-occupancies of 0.470 (6) and 0.530 (6) respectively.

In the crystal (Fig.2), molecules are linked *via* N2–H1N2···O3, N3–H1N3···O1, N3–H2N3···N1, O3–H1O3···O1, O3–H1O3···O2, C5–H5A···O1 and C16–H16A···O3 hydrogen bonds (Table 1) forming two-dimensional networks parallel to (010).

### Experimental

A mixture of 4- chloro-7- trifluoromethylquinoline (0.01 mole) and sulfanilamide (0.01 mole) in absolute ethanol (30 ml) was refluxed for 8h. The solid obtained was recrystallized from ethanol to give the title compound. Colourless plates were obtained by slow evaporation from a methanol/ethanol solvent mixture at room temperature.

### Refinement

Atoms H1N3 and H2N3 were located in a difference Fourier map and refined freely with N-H = 0.86 (3) and 0.88 (3) Å. Atom H1N2 was located in a difference Fourier map and was refined using a riding model, with *U*_iso_(H) = 1.3 *U*_eq_(N) [N-H = 0.8727]. The remaining hydrogen atoms were positioned geometrically [C–H = 0.93 or 0.96 Å, O–H = 0.95 Å] and were refined using a riding model, with *U*_iso_(H) = 1.2 *U*_eq_(C) or 1.5 *U*_eq_(C,O). Trifluoro atoms (F1/F2/F3) are disordered over two positions with refined site-occupancies of 0.56 (2) and 0.44 (2). A single solvate site is occupied at random by ethanol or methanol with refined site-occupancies of 0.470 (6) and 0.530 (6) respectively.

### Crystal data

**Table:**

C_16_H_12_F_3_N_3_O_2_S·0.47C_2_H_6_O·0.53CH_4_O	*Z* = 2
*M**_r_* = 405.98	*F*(000) = 420
Triclinic, *P*1	*D*_x_ = 1.472 Mg m^−^^3^
Hall symbol: -P 1	Cu *K*α radiation, λ = 1.54178 Å
*a* = 8.6037 (1) Å	Cell parameters from 2059 reflections
*b* = 9.3146 (2) Å	θ = 3.9–70.4°
*c* = 11.4590 (2) Å	µ = 2.07 mm^−^^1^
α = 92.463 (1)°	*T* = 296 K
β = 91.544 (1)°	Plate, colorless
γ = 92.969 (1)°	0.83 × 0.43 × 0.11 mm
*V* = 915.85 (3) Å^3^	

### Data collection

**Table:**

Bruker SMART APEXII CCD diffractometer	2845 independent reflections
Radiation source: fine-focus sealed tube	2631 reflections with *I* > 2σ(*I*)
Graphite monochromator	*R*_int_ = 0.026
φ and ω scans	θ_max_ = 63.0°, θ_min_ = 3.9°
Absorption correction: multi-scan (*SADABS*; Bruker, 2009)	*h* = −9→9
*T*_min_ = 0.279, *T*_max_ = 0.801	*k* = −10→10
9433 measured reflections	*l* = −12→10

### Refinement

**Table:**

Refinement on *F*^2^	Secondary atom site location: difference Fourier map
Least-squares matrix: full	Hydrogen site location: inferred from neighbouring sites
*R*[*F*^2^ > 2σ(*F*^2^)] = 0.045	H atoms treated by a mixture of independent and constrained refinement
*wR*(*F*^2^) = 0.129	*w* = 1/[σ^2^(*F*_o_^2^) + (0.0745*P*)^2^ + 0.2909*P*] where *P* = (*F*_o_^2^ + 2*F*_c_^2^)/3
*S* = 1.05	(Δ/σ)_max_ = 0.001
2845 reflections	Δρ_max_ = 0.34 e Å^−^^3^
309 parameters	Δρ_min_ = −0.41 e Å^−^^3^
2 restraints	Extinction correction: *SHELXL*, Fc^*^=kFc[1+0.001xFc^2^λ^3^/sin(2θ)]^-1/4^
Primary atom site location: structure-invariant direct methods	Extinction coefficient: 0.0031 (7)

### Special details

**Table:**

Geometry. All esds (except the esd in the dihedral angle between two l.s. planes) are estimated using the full covariance matrix. The cell esds are taken into account individually in the estimation of esds in distances, angles and torsion angles; correlations between esds in cell parameters are only used when they are defined by crystal symmetry. An approximate (isotropic) treatment of cell esds is used for estimating esds involving l.s. planes.
Refinement. Refinement of F^2^ against ALL reflections. The weighted R-factor wR and goodness of fit S are based on F^2^, conventional R-factors R are based on F, with F set to zero for negative F^2^. The threshold expression of F^2^ > 2sigma(F^2^) is used only for calculating R-factors(gt) etc. and is not relevant to the choice of reflections for refinement. R-factors based on F^2^ are statistically about twice as large as those based on F, and R- factors based on ALL data will be even larger.

### Fractional atomic coordinates and isotropic or equivalent isotropic displacement parameters (Å^2^)

**Table:**

	*x*	*y*	*z*	*U*_iso_*/*U*_eq_	Occ. (<1)
S1	0.13678 (6)	1.19048 (6)	0.59873 (4)	0.0525 (2)	
F1A	1.3524 (19)	0.588 (2)	1.0571 (10)	0.172 (6)	0.56 (2)
F2A	1.2936 (12)	0.4416 (8)	0.9214 (12)	0.116 (4)	0.56 (2)
F3A	1.4169 (11)	0.630 (2)	0.887 (2)	0.165 (5)	0.56 (2)
F1B	1.321 (2)	0.470 (2)	0.8836 (18)	0.154 (7)	0.44 (2)
F2B	1.2955 (16)	0.5212 (19)	1.0590 (12)	0.137 (5)	0.44 (2)
F3B	1.4139 (11)	0.6569 (10)	0.961 (2)	0.118 (4)	0.44 (2)
N1	0.8884 (2)	0.8734 (2)	1.08416 (15)	0.0558 (5)	
N2	0.6928 (3)	0.8826 (3)	0.74398 (17)	0.0723 (6)	
H1N2	0.7094	0.8068	0.6996	0.094*	
N3	−0.0014 (2)	1.1225 (3)	0.67275 (19)	0.0607 (5)	
O1	0.0977 (2)	1.15432 (19)	0.47846 (13)	0.0640 (4)	
O2	0.1626 (2)	1.33775 (18)	0.63484 (16)	0.0724 (5)	
C1	0.7685 (3)	0.9498 (3)	1.05917 (19)	0.0577 (6)	
H1A	0.7267	1.0026	1.1203	0.069*	
C2	0.6992 (2)	0.9581 (3)	0.94910 (19)	0.0545 (5)	
H2A	0.6143	1.0145	0.9388	0.065*	
C3	0.7565 (2)	0.8827 (2)	0.85519 (18)	0.0512 (5)	
C4	0.8899 (2)	0.8004 (2)	0.87623 (18)	0.0499 (5)	
C5	0.9674 (3)	0.7244 (3)	0.7879 (2)	0.0648 (6)	
H5A	0.9300	0.7250	0.7110	0.078*	
C6	1.0946 (3)	0.6508 (3)	0.8123 (2)	0.0680 (7)	
H6A	1.1438	0.6016	0.7527	0.082*	
C7	1.1524 (3)	0.6487 (3)	0.9279 (2)	0.0606 (6)	
C8	1.0800 (3)	0.7194 (2)	1.0154 (2)	0.0564 (5)	
H8A	1.1178	0.7155	1.0919	0.068*	
C9	0.9493 (2)	0.7981 (2)	0.99220 (18)	0.0489 (5)	
C10	1.3000 (3)	0.5751 (3)	0.9526 (3)	0.0769 (8)	
C11	0.5640 (3)	0.9600 (3)	0.70824 (19)	0.0596 (6)	
C12	0.5560 (3)	1.1052 (3)	0.7346 (2)	0.0616 (6)	
H12A	0.6380	1.1554	0.7756	0.074*	
C13	0.4265 (3)	1.1765 (3)	0.7003 (2)	0.0578 (6)	
H13A	0.4199	1.2738	0.7203	0.069*	
C14	0.3073 (2)	1.1028 (2)	0.63654 (18)	0.0504 (5)	
C15	0.3168 (3)	0.9587 (3)	0.6061 (2)	0.0671 (7)	
H15A	0.2377	0.9102	0.5609	0.081*	
C16	0.4443 (3)	0.8871 (3)	0.6433 (2)	0.0718 (7)	
H16A	0.4499	0.7893	0.6246	0.086*	
H2N3	0.020 (3)	1.130 (3)	0.746 (3)	0.061 (7)*	
H1N3	−0.034 (3)	1.037 (3)	0.645 (3)	0.075 (9)*	
O3	0.6404 (7)	0.5947 (6)	0.6144 (5)	0.120 (2)	0.530 (6)
H1O3	0.7187	0.6422	0.5695	0.181*	0.530 (6)
C17	0.7032 (11)	0.4787 (8)	0.6295 (13)	0.148 (4)	0.530 (6)
H17A	0.7984	0.4949	0.6749	0.223*	0.530 (6)
H17B	0.6311	0.4193	0.6712	0.223*	0.530 (6)
H17C	0.7243	0.4313	0.5560	0.223*	0.530 (6)
C18	0.3657 (7)	0.5101 (10)	0.4490 (6)	0.084 (2)	0.470 (6)
H18A	0.4198	0.5162	0.3770	0.100*	0.470 (6)
H18B	0.3355	0.4103	0.4562	0.100*	0.470 (6)
C19	0.2268 (12)	0.5869 (11)	0.4363 (12)	0.131 (4)	0.470 (6)
H19A	0.1698	0.5435	0.3695	0.196*	0.470 (6)
H19B	0.1649	0.5786	0.5043	0.196*	0.470 (6)
H19C	0.2508	0.6867	0.4235	0.196*	0.470 (6)
O4	0.4659 (8)	0.5410 (5)	0.5282 (6)	0.130 (3)	0.470 (6)
H1O4	0.5659	0.5073	0.5128	0.195*	0.470 (6)

### Atomic displacement parameters (Å^2^)

**Table:**

	*U*^11^	*U*^22^	*U*^33^	*U*^12^	*U*^13^	*U*^23^
S1	0.0516 (3)	0.0669 (4)	0.0399 (3)	0.0193 (2)	−0.0105 (2)	0.0026 (2)
F1A	0.138 (8)	0.260 (12)	0.120 (7)	0.136 (8)	−0.081 (6)	−0.078 (7)
F2A	0.087 (3)	0.065 (3)	0.196 (12)	0.027 (3)	−0.023 (4)	0.014 (5)
F3A	0.059 (3)	0.210 (10)	0.243 (12)	0.052 (4)	0.031 (5)	0.124 (9)
F1B	0.152 (11)	0.182 (13)	0.128 (8)	0.127 (10)	−0.068 (7)	−0.080 (8)
F2B	0.103 (6)	0.187 (9)	0.132 (9)	0.069 (6)	−0.014 (5)	0.083 (9)
F3B	0.044 (3)	0.105 (4)	0.205 (12)	0.005 (3)	−0.022 (6)	0.029 (6)
N1	0.0571 (11)	0.0718 (11)	0.0393 (10)	0.0096 (9)	−0.0032 (8)	0.0045 (8)
N2	0.0639 (12)	0.1111 (17)	0.0443 (11)	0.0455 (12)	−0.0132 (9)	−0.0094 (10)
N3	0.0517 (11)	0.0880 (16)	0.0435 (12)	0.0193 (10)	−0.0071 (8)	0.0019 (10)
O1	0.0681 (10)	0.0856 (11)	0.0397 (9)	0.0222 (8)	−0.0117 (7)	0.0052 (7)
O2	0.0794 (12)	0.0654 (10)	0.0728 (11)	0.0213 (8)	−0.0194 (9)	−0.0012 (8)
C1	0.0546 (13)	0.0764 (14)	0.0431 (12)	0.0127 (10)	0.0047 (9)	0.0018 (10)
C2	0.0429 (11)	0.0743 (14)	0.0478 (12)	0.0140 (9)	0.0029 (8)	0.0068 (10)
C3	0.0403 (10)	0.0718 (13)	0.0424 (11)	0.0126 (9)	−0.0032 (8)	0.0047 (9)
C4	0.0434 (11)	0.0641 (12)	0.0428 (11)	0.0090 (9)	−0.0035 (8)	0.0037 (9)
C5	0.0629 (14)	0.0883 (17)	0.0447 (13)	0.0271 (12)	−0.0072 (10)	−0.0008 (11)
C6	0.0639 (15)	0.0850 (16)	0.0571 (14)	0.0314 (12)	−0.0046 (11)	−0.0039 (11)
C7	0.0502 (12)	0.0637 (13)	0.0683 (15)	0.0145 (10)	−0.0126 (10)	0.0030 (11)
C8	0.0536 (12)	0.0642 (13)	0.0510 (13)	0.0072 (10)	−0.0156 (10)	0.0052 (10)
C9	0.0448 (11)	0.0595 (11)	0.0423 (11)	0.0032 (9)	−0.0060 (8)	0.0065 (8)
C10	0.0619 (17)	0.0797 (18)	0.090 (2)	0.0252 (14)	−0.0209 (14)	0.0023 (15)
C11	0.0482 (12)	0.0918 (17)	0.0405 (12)	0.0249 (11)	−0.0059 (9)	0.0021 (10)
C12	0.0479 (12)	0.0813 (16)	0.0554 (14)	0.0065 (10)	−0.0125 (10)	0.0054 (11)
C13	0.0528 (12)	0.0680 (13)	0.0529 (13)	0.0087 (10)	−0.0089 (9)	0.0061 (10)
C14	0.0463 (11)	0.0670 (13)	0.0391 (11)	0.0158 (9)	−0.0059 (8)	0.0046 (9)
C15	0.0599 (14)	0.0769 (16)	0.0637 (15)	0.0210 (11)	−0.0241 (11)	−0.0112 (12)
C16	0.0725 (16)	0.0784 (16)	0.0649 (16)	0.0330 (13)	−0.0241 (12)	−0.0127 (12)
O3	0.144 (5)	0.093 (3)	0.119 (4)	0.004 (3)	−0.041 (3)	−0.016 (3)
C17	0.111 (8)	0.139 (10)	0.191 (13)	−0.020 (7)	−0.039 (8)	0.017 (8)
C18	0.068 (4)	0.124 (7)	0.065 (4)	0.050 (4)	0.002 (3)	0.026 (4)
C19	0.104 (7)	0.090 (6)	0.203 (12)	0.024 (5)	0.006 (7)	0.034 (6)
O4	0.160 (6)	0.071 (3)	0.159 (7)	0.004 (3)	0.039 (5)	−0.022 (3)

### Geometric parameters (Å, º)

**Table:**

S1—O2	1.4202 (18)	C7—C10	1.500 (3)
S1—O1	1.4314 (16)	C8—C9	1.399 (3)
S1—N3	1.599 (2)	C8—H8A	0.9300
S1—C14	1.768 (2)	C11—C12	1.379 (4)
F1A—C10	1.266 (8)	C11—C16	1.385 (4)
F2A—C10	1.277 (8)	C12—C13	1.383 (3)
F3A—C10	1.362 (9)	C12—H12A	0.9300
F1B—C10	1.258 (10)	C13—C14	1.379 (3)
F2B—C10	1.339 (10)	C13—H13A	0.9300
F3B—C10	1.209 (9)	C14—C15	1.379 (3)
N1—C1	1.316 (3)	C15—C16	1.381 (3)
N1—C9	1.371 (3)	C15—H15A	0.9300
N2—C3	1.373 (3)	C16—H16A	0.9300
N2—C11	1.414 (3)	O3—C17	1.2497 (11)
N2—H1N2	0.8727	O3—H1O3	0.9600
N3—H2N3	0.86 (3)	C17—H17A	0.9600
N3—H1N3	0.88 (3)	C17—H17B	0.9600
C1—C2	1.387 (3)	C17—H17C	0.9600
C1—H1A	0.9300	C18—O4	1.2485 (11)
C2—C3	1.376 (3)	C18—C19	1.432 (11)
C2—H2A	0.9300	C18—O4^i^	1.567 (11)
C3—C4	1.434 (3)	C18—H18A	0.9600
C4—C5	1.412 (3)	C18—H18B	0.9600
C4—C9	1.412 (3)	C19—H19A	0.9600
C5—C6	1.350 (3)	C19—H19B	0.9600
C5—H5A	0.9300	C19—H19C	0.9600
C6—C7	1.404 (3)	O4—O4^i^	1.171 (11)
C6—H6A	0.9300	O4—C18^i^	1.567 (11)
C7—C8	1.359 (4)	O4—H1O4	0.9500
			
O2—S1—O1	118.81 (11)	F3B—C10—C7	113.3 (5)
O2—S1—N3	108.50 (13)	F1B—C10—C7	114.0 (6)
O1—S1—N3	106.49 (12)	F1A—C10—C7	115.9 (4)
O2—S1—C14	107.14 (11)	F2A—C10—C7	114.0 (5)
O1—S1—C14	108.12 (10)	F2B—C10—C7	109.6 (5)
N3—S1—C14	107.28 (10)	F3A—C10—C7	110.3 (4)
C1—N1—C9	116.18 (18)	C12—C11—C16	119.6 (2)
C3—N2—C11	126.10 (19)	C12—C11—N2	121.9 (2)
C3—N2—H1N2	115.2	C16—C11—N2	118.5 (2)
C11—N2—H1N2	114.7	C11—C12—C13	120.2 (2)
S1—N3—H2N3	111.6 (17)	C11—C12—H12A	119.9
S1—N3—H1N3	112 (2)	C13—C12—H12A	119.9
H2N3—N3—H1N3	116 (3)	C14—C13—C12	119.8 (2)
N1—C1—C2	125.4 (2)	C14—C13—H13A	120.1
N1—C1—H1A	117.3	C12—C13—H13A	120.1
C2—C1—H1A	117.3	C13—C14—C15	120.4 (2)
C3—C2—C1	119.9 (2)	C13—C14—S1	120.16 (17)
C3—C2—H2A	120.0	C15—C14—S1	119.35 (17)
C1—C2—H2A	120.0	C14—C15—C16	119.6 (2)
N2—C3—C2	123.6 (2)	C14—C15—H15A	120.2
N2—C3—C4	119.04 (19)	C16—C15—H15A	120.2
C2—C3—C4	117.38 (19)	C15—C16—C11	120.3 (2)
C5—C4—C9	118.1 (2)	C15—C16—H16A	119.8
C5—C4—C3	124.07 (19)	C11—C16—H16A	119.8
C9—C4—C3	117.80 (19)	C17—O3—H1O3	99.6
C6—C5—C4	121.6 (2)	O3—C17—H17A	110.6
C6—C5—H5A	119.2	O3—C17—H17B	106.9
C4—C5—H5A	119.2	H17A—C17—H17B	109.5
C5—C6—C7	119.9 (2)	O3—C17—H17C	110.9
C5—C6—H6A	120.1	H17A—C17—H17C	109.5
C7—C6—H6A	120.1	H17B—C17—H17C	109.5
C8—C7—C6	120.2 (2)	O4—C18—C19	122.6 (9)
C8—C7—C10	120.4 (2)	O4—C18—O4^i^	47.5 (5)
C6—C7—C10	119.3 (2)	C19—C18—O4^i^	167.5 (9)
C7—C8—C9	121.1 (2)	O4—C18—H18A	105.7
C7—C8—H8A	119.5	C19—C18—H18A	107.2
C9—C8—H8A	119.5	O4^i^—C18—H18A	72.6
N1—C9—C8	117.50 (19)	O4—C18—H18B	106.1
N1—C9—C4	123.32 (19)	C19—C18—H18B	107.9
C8—C9—C4	119.1 (2)	O4^i^—C18—H18B	83.8
F3B—C10—F1B	111.2 (9)	H18A—C18—H18B	106.3
F3B—C10—F1A	69.1 (7)	C18—C19—H19A	106.8
F1B—C10—F1A	124.4 (7)	C18—C19—H19B	110.5
F3B—C10—F2A	127.6 (7)	H19A—C19—H19B	109.5
F1A—C10—F2A	107.9 (8)	C18—C19—H19C	111.1
F3B—C10—F2B	102.5 (7)	H19A—C19—H19C	109.5
F1B—C10—F2B	105.4 (9)	H19B—C19—H19C	109.5
F2A—C10—F2B	81.8 (8)	O4^i^—O4—C18	80.7 (6)
F1B—C10—F3A	78.6 (8)	O4^i^—O4—C18^i^	51.8 (4)
F1A—C10—F3A	105.2 (7)	C18—O4—C18^i^	132.5 (5)
F2A—C10—F3A	102.4 (9)	C18—O4—H1O4	114.4
F2B—C10—F3A	133.7 (6)		
			
C9—N1—C1—C2	0.9 (3)	C8—C7—C10—F1A	−2.1 (15)
N1—C1—C2—C3	−0.2 (4)	C6—C7—C10—F1A	174.3 (14)
C11—N2—C3—C2	1.4 (4)	C8—C7—C10—F2A	124.1 (7)
C11—N2—C3—C4	−178.6 (2)	C6—C7—C10—F2A	−59.5 (7)
C1—C2—C3—N2	178.6 (2)	C8—C7—C10—F2B	34.5 (10)
C1—C2—C3—C4	−1.4 (3)	C6—C7—C10—F2B	−149.1 (10)
N2—C3—C4—C5	3.9 (4)	C8—C7—C10—F3A	−121.4 (13)
C2—C3—C4—C5	−176.1 (2)	C6—C7—C10—F3A	55.0 (13)
N2—C3—C4—C9	−177.6 (2)	C3—N2—C11—C12	50.6 (4)
C2—C3—C4—C9	2.3 (3)	C3—N2—C11—C16	−130.4 (3)
C9—C4—C5—C6	0.2 (4)	C16—C11—C12—C13	2.5 (4)
C3—C4—C5—C6	178.7 (2)	N2—C11—C12—C13	−178.6 (2)
C4—C5—C6—C7	0.1 (4)	C11—C12—C13—C14	−2.0 (4)
C5—C6—C7—C8	0.6 (4)	C12—C13—C14—C15	−0.4 (4)
C5—C6—C7—C10	−175.8 (3)	C12—C13—C14—S1	176.73 (17)
C6—C7—C8—C9	−1.5 (4)	O2—S1—C14—C13	5.9 (2)
C10—C7—C8—C9	174.9 (2)	O1—S1—C14—C13	135.11 (19)
C1—N1—C9—C8	178.0 (2)	N3—S1—C14—C13	−110.4 (2)
C1—N1—C9—C4	0.2 (3)	O2—S1—C14—C15	−176.9 (2)
C7—C8—C9—N1	−176.2 (2)	O1—S1—C14—C15	−47.8 (2)
C7—C8—C9—C4	1.7 (3)	N3—S1—C14—C15	66.7 (2)
C5—C4—C9—N1	176.8 (2)	C13—C14—C15—C16	2.2 (4)
C3—C4—C9—N1	−1.8 (3)	S1—C14—C15—C16	−175.0 (2)
C5—C4—C9—C8	−1.0 (3)	C14—C15—C16—C11	−1.6 (4)
C3—C4—C9—C8	−179.6 (2)	C12—C11—C16—C15	−0.7 (4)
C8—C7—C10—F3B	−79.2 (13)	N2—C11—C16—C15	−179.7 (2)
C6—C7—C10—F3B	97.2 (13)	C19—C18—O4—O4^i^	−170.3 (9)
C8—C7—C10—F1B	152.4 (15)	C19—C18—O4—C18^i^	−170.3 (9)
C6—C7—C10—F1B	−31.2 (16)	O4^i^—C18—O4—C18^i^	−0.003 (1)

Symmetry code: (i) −*x*+1, −*y*+1, −*z*+1.

### Hydrogen-bond geometry (Å, º)

**Table:**

*D*—H···*A*	*D*—H	H···*A*	*D*···*A*	*D*—H···*A*
N2—H1*N*2···O3	0.87	2.21	3.016 (6)	153
N3—H2*N*3···N1^ii^	0.85 (3)	2.08 (3)	2.924 (3)	169 (3)
N3—H1*N*3···O1^iii^	0.87 (3)	2.26 (3)	3.107 (3)	163 (3)
O3—H1*O*3···O1^iv^	0.96	2.49	3.387 (6)	155
O3—H1*O*3···O2^iv^	0.96	2.59	3.425 (6)	146
C5—H5*A*···O1^iv^	0.93	2.50	3.343 (3)	151
C16—H16*A*···O3	0.93	2.51	3.287 (6)	141

Symmetry codes: (ii) −*x*+1, −*y*+2, −*z*+2; (iii) −*x*, −*y*+2, −*z*+1; (iv) −*x*+1, −*y*+2, −*z*+1.
